# Detecting genes associated with antimicrobial resistance and pathogen virulence in three New Zealand rivers

**DOI:** 10.7717/peerj.12440

**Published:** 2021-12-03

**Authors:** Meredith Davis, Anne C. Midwinter, Richard Cosgrove, Russell G. Death

**Affiliations:** 1 School of Agriculture and the Environment, Massey University, Palmerston North, Manawatu, New Zealand; 2 Molecular Epidemiology and Veterinary Public Health Laboratory - Hopkirk Research Institute, School of Veterinary Science, Massey University, Palmerston North, Manawatu, New Zealand; 3 Fish and Game New Zealand, Temuka, Canterbury, New Zealand

**Keywords:** Antimicrobial resistance (AMR), *E. coli*, eDNA, Pathogen, Water quality, Zoonoses

## Abstract

The emergence of clinically significant antimicrobial resistance (AMR) in bacteria is frequently attributed to the use of antimicrobials in humans and livestock and is often found concurrently with human and animal pathogens. However, the incidence and natural drivers of antimicrobial resistance and pathogenic virulence in the environment, including waterways and ground water, are poorly understood. Freshwater monitoring for microbial pollution relies on culturing bacterial species indicative of faecal pollution, but detection of genes linked to antimicrobial resistance and/or those linked to virulence is a potentially superior alternative. We collected water and sediment samples in the autumn and spring from three rivers in Canterbury, New Zealand; sites were above and below reaches draining intensive dairy farming. Samples were tested for loci associated with the AMR-related group 1 CTX-M enzyme production (*bla*_CTX-M_) and Shiga toxin producing *Escherichia coli* (STEC). The *bla*_CTX-M_ locus was only detected during spring and was more prevalent downstream of intensive dairy farms. Loci associated with STEC were detected in both the autumn and spring, again predominantly downstream of intensive dairying. This cross-sectional study suggests that targeted testing of environmental DNA is a useful tool for monitoring waterways. Further studies are now needed to extend our observations across seasons and to examine the relationship between the presence of these genetic elements and the incidence of disease in humans.

## Introduction

Waterborne diseases are a significant threat to public health and the majority of them are zoonotic (*e.g*., hosted by animals) (Cleaveland, Laurenson & Taylor, 2001; World Health Organization, 2011). There is a growing body of evidence that intensive livestock farming degrades freshwater ecosystems and has the potential to create stores of zoonotic bacteria in rivers (Monaghan, de Klein & Muirhead, 2008; Daszak et al., 2020; Phiri et al., 2020). Environmental degradation is a common precursor to infectious disease emergence (Pimentel et al., 2007; Daszak et al., 2020). Therefore, understanding the role of catchment land use and recreation in waterways in the spread of waterborne zoonoses has become even more critical.

Antimicrobial resistant infectious diseases are emerging as one of the most pressing global public health issues (Hernando-Amado et al., 2019). Antimicrobial resistance (AMR) is a result of interactions between microbes and their environment, both biotic and abiotic; the predominant driver is considered the use of antibiotics in livestock and humans (Hernando-Amado et al., 2019). Patterns of AMR emergence are non-random; however, our ability to predict emergence is limited because it involves interactions between multiple drivers and non-linear patterns associated with human systems (Daszak, Cunningham & Hyatt, 2000; Jones et al., 2008). These systems are best investigated using a One Health approach, which considers human, animal, and environmental health as interconnected, providing a useful framework for addressing the emergence and spread of both AMR and zoonotic diseases (Rubin et al., 2013; Harrison et al., 2020).

Enterobacteriaceae—including *Escherichia coli*—are recognized as an important group of bacteria that carry AMR in clinical settings (Pendleton, Gorman & Gilmore, 2013). Until recently AMR bacteria were primarily of interest in hospital settings (Cosgrove, 2006; Bancroft, 2007), but they have increasingly been identified in the environment (Wyres & Holt, 2018), wildlife (Wilharm et al., 2017) and domestic animals (Singh, 2018). The bovine rumen is also a known reservoir for AMR bacteria (Auffret et al., 2017). Bacteria residing in the digestive tract are often impacted by antibiotic use and then, in pastoral farming systems, excreted onto land where they are potentially transported into waterways (Kirchner et al., 2013; Dwivedi, Mohanty & Lesikar, 2016).

The extended spectrum β-lactamases (ESBL) enzymes provide resistance to beta-lactam antibiotics, including first and third generation cephalosporins, carbapenems and penicillins (Bonnet, 2004). The production of ESBLs is an emerging and spreading AMR (Lewis et al., 2007). Critically, many of the genes encoding β-lactamase production can be horizontally transferred *via* plasmids (Carattoli, 2013). Some ESBLs, specifically the group 1 CTX-M β-lactamases, are recurrently co-morbid with zoonotic pathogens (*e.g*., Shiga toxin producing *Escherichia coli*–STEC–(Ishii et al., 2005; Valat et al., 2012), a group of human pathogens commonly hosted by cows (Oporto et al., 2019)). STEC are an important group of zoonoses by themselves; the fourth most reported zoonotic disease in the EU (European Center for Disease Prevention & Control, 2018) and USA (Centers for Disease Control & Prevention, 2018) and increasing in prevalence in New Zealand (Public Health Surveillance: Environment Group ESR, 2019). STEC have been found in recreational waters and are transmitted *via* the oral-faecal route (Ahmed, Gyawali & Toze, 2015; Swaggerty et al., 2018).

Molecular characterization is routinely used to classify cultured bacterial isolates, as culturing alone is unable to identify all the genetic components imparting AMR or virulence (Ram et al., 2009). Pre-isolation culturing commonly uses an enrichment step prior to molecular characterization which has been found to affect plasmid retention (reportedly up to a 95% loss) and may select against the strains being sought (Hill & Carlisle, 1981; Sowers, Wells & Strockbine, 1996; Wein et al., 2019). However, an alternative is to test environmental DNA (eDNA) directly. This offers a rapid, inexpensive survey of specific genetic elements chosen for their relevance to environmental, animal, or human health, such as loci associated with AMR and/or virulence. Although, this approach does not yield individual pathogenic/AMR colony isolates, it does provide an indication of whether the relevant genetic elements are present and whether further investigation is warranted. Testing samples for genetic loci associated with human pathogens is commonly employed in screening food and has been used in waterways internationally; however, this is a novel variation of those methods and new in New Zealand (Fraiture et al., 2020; Haberecht et al., 2019; Heijnen & Medema, 2006; Koutsoumanis et al., 2020).

In this case study we tested eDNA collected from sites on three Canterbury, New Zealand rivers during autumn (May) and spring (September), which coincides with seasonal peaks in human STEC cases (Public Health Surveillance: Environment Group ESR, 2019). We evaluated both benthic sediments and water column samples for seven genes; one, a general indicator of *E*. *coli* (*uidA*), with the remaining six frequently associated with group 1 CTX-M β-lactamase production (*bla*_CTX-M_) and human-pathogenic STEC virulence (*stx*_*1*_, *stx*_*2*_ and *eae*) and STEC serotype (O157 *rfbE* and O26 *wzy*).

## Materials & methods

### Sample collection

**Figure 1:**
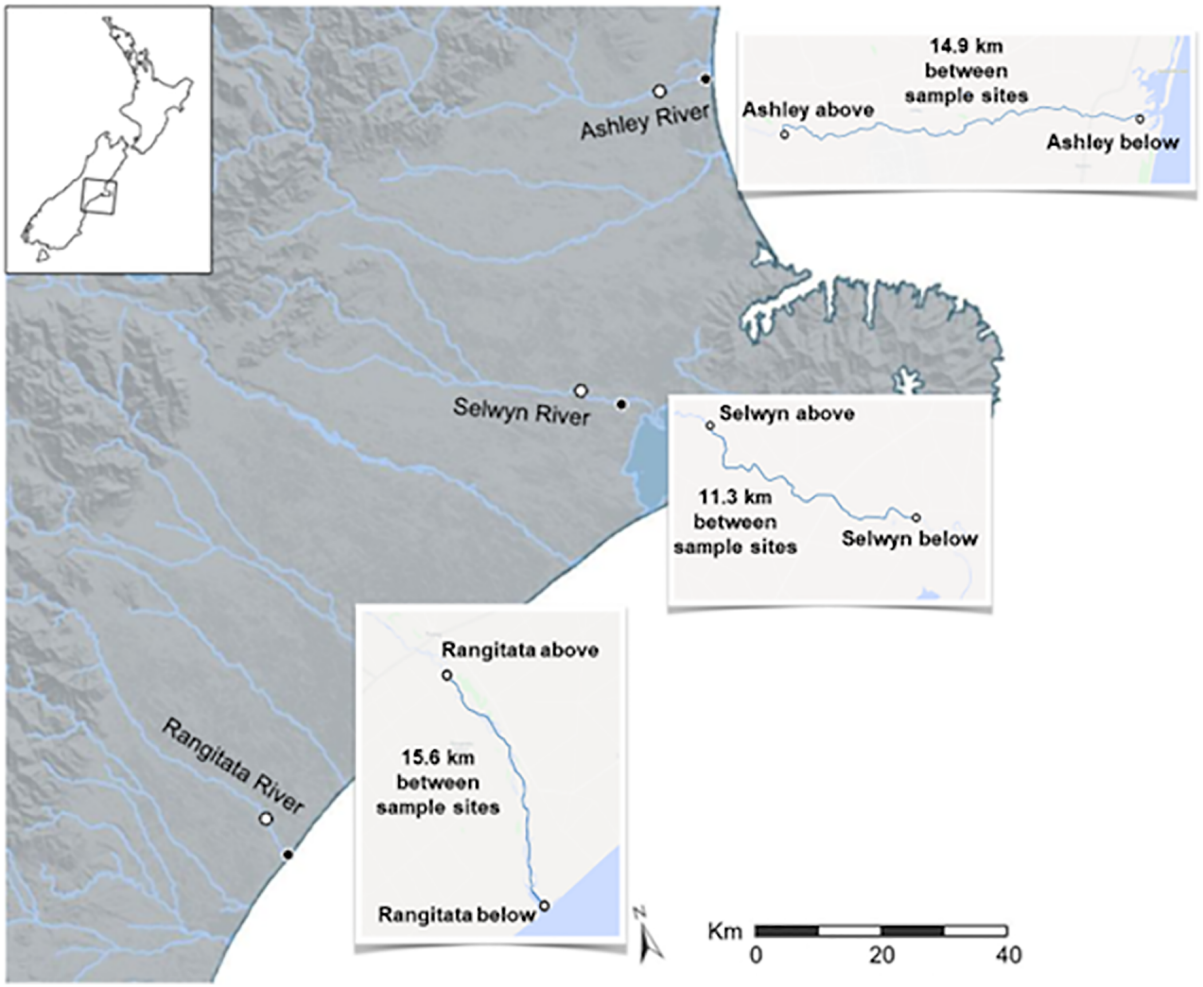
Map of the three Canterbury rivers. Map of central and southern Canterbury, New Zealand with the Ashley, Rangitata, and Selwyn rivers labelled. Locations at which water and sediment were collected during May and September 2018 are marked with circles; for each river the white centered dot indicates the ‘above intensive dairy’ site and the black centered dot the ‘below intensive dairy’ site.

Two substrates, water and sediment, were sampled, collected from the Ashley, Rangitata, and Selwyn rivers once in austral autumn and spring, 2018. Collections were made at two sites along each river; these sites were 10–15 km apart with one above and the other below reaches draining intensive dairy farms (Fig. 1). At each site, water and benthic sediment samples were collected in separate sterile containers. Three 1 L water and three 25 g sediment samples were collected at each site. Samples were packed on ice, transported to the laboratory, and processed within 24 h of collection.

### Sample processing for bacterial culturing

Water column sample aliquots were diluted 1:10, 1:5, and 1:2.5 with sterile MilliQ H_2_O to a final volume of 50 ml. At sites where the level of suspended sediments was high, an additional 1:50 dilution was also prepared to ensure ease of enumeration. For each sample we prepared three technical replicates at each dilution (*i.e*., 9–12 dilutions per sample).

For sediment samples, 2 g of wet sediment was first transferred to a 5 ml microtube, 3 ml MilliQ H_2_O added, and the mixture then agitated vigorously for 30 s. Aliquots of the resulting supernatant were diluted 1:500, 1:200, 1:100, and 1:50 with MilliQ H_2_O to a final volume of 50 ml. For each sample we prepared three technical replicates at each dilution (*i.e*., 12 dilutions per sample).

For each dilution the total 50 ml volume was vacuum filtered through a single sterile 0.45 µm cellulose ester membrane filter (Merck KGaA, Darmstadt, Germany).

### Bacterial culturing

Bacterial culturing followed United States Environmental Protection Agency method 1603 (United States Environmental Protection Agency (US EPA), 2015). Each filter was placed onto a Difco Modified mTEC Agar (VWR, Radnor, PA, USA) plate, incubated at 37.5 °C for 2 h, and then incubated at 45 °C for 18–20 h. Following incubation, colonies indicative of *E. coli* (red/magenta colonies) were counted.

### Sample processing for molecular testing

Three 500 ml aliquots of water from each site were vacuum filtered through separate 0.45 µm cellulose ester membrane filters. Environmental DNA was extracted from half of each filter using the NucleoSpin® soil kit (Machery-Nagel GmbH and Co. KG, Düren, Germany) following the manufacturer’s instructions. For each sediment sample eDNA was extracted from three 0.5 g aliquots of wet sediment, using the NucleoSpin® soil kit.

### Molecular testing for target genes

We evaluated the presence of antimicrobial resistance using a polymerase chain reaction (PCR) targeting the *bla* gene associated with group 1 CTX-M β-lactamases (*bla*_CTX-M_) using the primers of Lalzampuia et al. (2013). Molecular characterization of STEC focused on genes associated with virulence. Specifically, we targeted genes associated with serogroup specific antigen biosynthesis, *rfbE* for O157 and *wzy* for O26, the *stx*_1_ and *stx*_2_ toxin genes, and the intimate attachment and effacing gene, *eae*, using the primers reported by Anklam et al. (2012). The detection limits of the STEC assays have been reported at 10^3^ CFU/ml (Anklam et al., 2012) with a specificity to sensitivity ratio at 95:92% for O157 strains 92:91% for O26 strains (*eae*, *stx*_*1*_, and *stx*_*2*_ genes inclusive) (Browne et al., 2018). As an amplification control, we targeted the beta-glucuronidase gene, *uidA*, which is present in most *E*. *coli* (Table 1) (Anklam et al., 2012).

**Table 1:** Details of the oligonucleotide primers used in this study.

Gene	Primer sequences	Product size	Refs
*bla*_CTX-M_	Forward: 5′ CCCATGGTTAAAAAACACTGC-3′ Reverse: 5′ CAGCGCTTTTGCCGTCTAAG-3′	950 bp	(Lalzampuia et al., 2013)
*uidA*	Forward: 5′ AGTGTGATATCTACCCGCTT-3′ Reverse: 5′ AGAACGGTTTGTGGTTAATCAG-3′	84 bp	(Anklam et al., 2012)
*stx*_*1*_	Forward: 5′ GGATAATTTGTTTGCAGTTGATGTC-3′ Reverse: 5′ CAAATCCTGTCACATATAAATTATTTCGT-3′	107 bp	(Anklam et al., 2012)
*stx*_*2*_	Forward: 5′ GGGCAGTTATTTTGCTGTGGA-3′ Reverse: 5′ GAAAGTATTTGTTGCCGTATTAACGA-3′	131 bp	(Anklam et al., 2012)
*eae*	Forward: 5′ CATTGATCAGGATTTTTCTGGTGATA-3′ Reverse: 5′ CTCATGCGGAAATAGCCGTTA-3′	102 bp	(Anklam et al., 2012)
*wzy* O26	Forward: 5′AGCGTATGTTGATATATTTAATGTC-3′ Reverse: 5′AATGTGGTCCCAAGGAATAAA-3′	141 bp	(Anklam et al., 2012)

Amplification reactions were performed in 20 μl reaction volumes containing 0.5 × iQ PerfeCTa® qPCR ToughMix™, ROX™ (QIAGEN, Düsseldorf, Germany), 1 pM of each primer, and 2.5 μl of DNA template. Thermocycling was performed in a T1 thermocycler (Biometra GmbH, Göttingen, Germany) using standard cycling conditions including an initial denaturation at 94 °C for 3 min, followed by 35 cycles of 94 °C for 30 s, 60 °C for 30 s and 72 °C for 1 min, with a final extension at 72 °C for 5 min. Amplification products were visualized using SYBR Safe (ThermoFisher Scientific, Waltham, MA, USA) following electrophoresis in 2% Tris-acetate-ethylenediamine tetraacetic acid agarose gels.

## Results

### Bacterial culturing

With one exception, (the spring sampling at the ‘above intensive dairy’ site on the Selwyn River), colonies indicative of *E*. *coli* were consistently higher from sediment than water column samples (Table 2). Moreover, counts were also higher for all but one sample taken at the sites ‘below intensive dairy’ sites. Only the spring sediment sample from the site ‘above intensive dairy’ on the Rangitata River had a more *E*. *coli* than the corresponding sample from the site ‘below intensive dairy’.

**Table 2:** Presumptive *Escherichia coli* colony counts, in CFU/100 ml, for water and sediment samples collected from the Selwyn, Rangitata, and Ashley rivers during May and September 2018.

Sampling time	Sampling sites and substrates											
	‘above intensive dairy’						‘below intensive dairy’					
	Ashley water	Ashley sediment	Rangitata water	Rangitata sediment	Selwyn water	Selwyn sediment	Ashley water	Ashley sediment	Rangitata water	Rangitata sediment	Selwyn water	Selwyn sediment
May (autumn)	45	230	20	7,100	10	200	440	730	175	273,300	160	230
September (spring)	20	400	20	5,000	505	200	2,000	3,600	40	1,600	2,250	12,200

### Molecular testing for target genes

The *uidA* locus was successfully amplified from every sample (Table 3). In contrast, detection of the gene associated with antibiotic resistance (*bla*_CTX-M_) and the three gene loci associated with pathogenic STEC (*stx*_*1*_, *stx*_*2*_ and *eae*) varied with location, time and substrate. The antibiotic resistance gene was detected in both substrates, with equal frequency, in samples from the Selwyn and Rangitata rivers. The *stx*_*1*_, *stx*_*2*_ and *eae* genes were more frequently detected in water samples in the autumn but in sediment samples in the spring. In both the autumn and spring, serotype genes (O26 *wzy* and O157 *rfbE*) were amplified from the same sample as one or more toxin genes (*stx*_*1*_ or *stx*_*2*_) and the effacement gene (*eae*). The O26 marker was more frequently recovered from water, while that for O157 was more common in sediment.

**Table 3:** Presence of *Escherichia coli* control (*uidA*), STEC virulence (*stx_1_*, *stx_2_*, *eae*), serogroup (O26 *wzy* and O157 *rfbE*), and antibiotic resistance (*bla*_CTX-M_) genes in water and sediment samples collected from the Selwyn, Rangitata, and Ashley rivers during May and September 2018.

Gene locus	Sampling sites and substrates											
	‘above intensive dairy’						‘below intensive dairy’					
	Ashley water	Ashley sediment	Rangitata water	Rangitata sediment	Selwyn water	Selwyn sediment	Ashley water	Ashley sediment	Rangitata water	Rangitata sediment	Selwyn water	Selwyn sediment
May (autumn)												
*uidA*	+	+	+	+	+	+	+	+	+	+	+	+
*stx*_*1*_	−	−	−	−	−	−	+	+	+	−	−	−
*stx*_*2*_	−	−	−	−	−	−	−	+	+	−	−	−
*eae*	−	−	−	−	−	−	+	+	+	−	−	−
*wzy* O26	−	−	−	−	−	−	+	+	+	−	−	−
*rfbE* O157	−	−	−	−	−	+	+	+	+	−	−	+
*bla*_CTX-M_	−	−	−	−	−	−	−	−	−	−	−	−
September (spring)												
*uidA*	+	+	+	+	+	+	+	+	+	+	+	+
*stx*_*1*_	−	+	−	+	−	−	−	−	+	−	+	+
*stx*_*2*_	−	+	−	+	−	+	+	−	−	+	−	+
*eae*	−	+	+	+	−	+	+	+	+	+	+	+
*wzy* 026	−	−	−	−	−	−	−	−	+	−	−	−
*rfbE* O157	−	−	−	−	−	+	−	−	−	+	+	+
*bla*_CTX-M_	−	−	−	+	−	−	−	−	+	−	+	+

In autumn, the genes associated with pathogenic STEC O157 (*i.e*., *stx*_*1*_, *stx*_*2*_, *eae* and *rfbE*) were present in 25% of the samples. All three virulence and both serogroup genes were detected in the autumn, both substrates from the Ashley and the water from the Rangitata had both serotype markers in the ‘below intensive dairy’ samples. Additionally, the *stx*_*1*_, *eae*, and *rfbE* genes were present in water from the site ‘below intensive dairy’ on the Ashley River in spring. The *bla*_CTX-M_ gene associated with AMR was not detected in any autumn samples.

**Figure 2:**
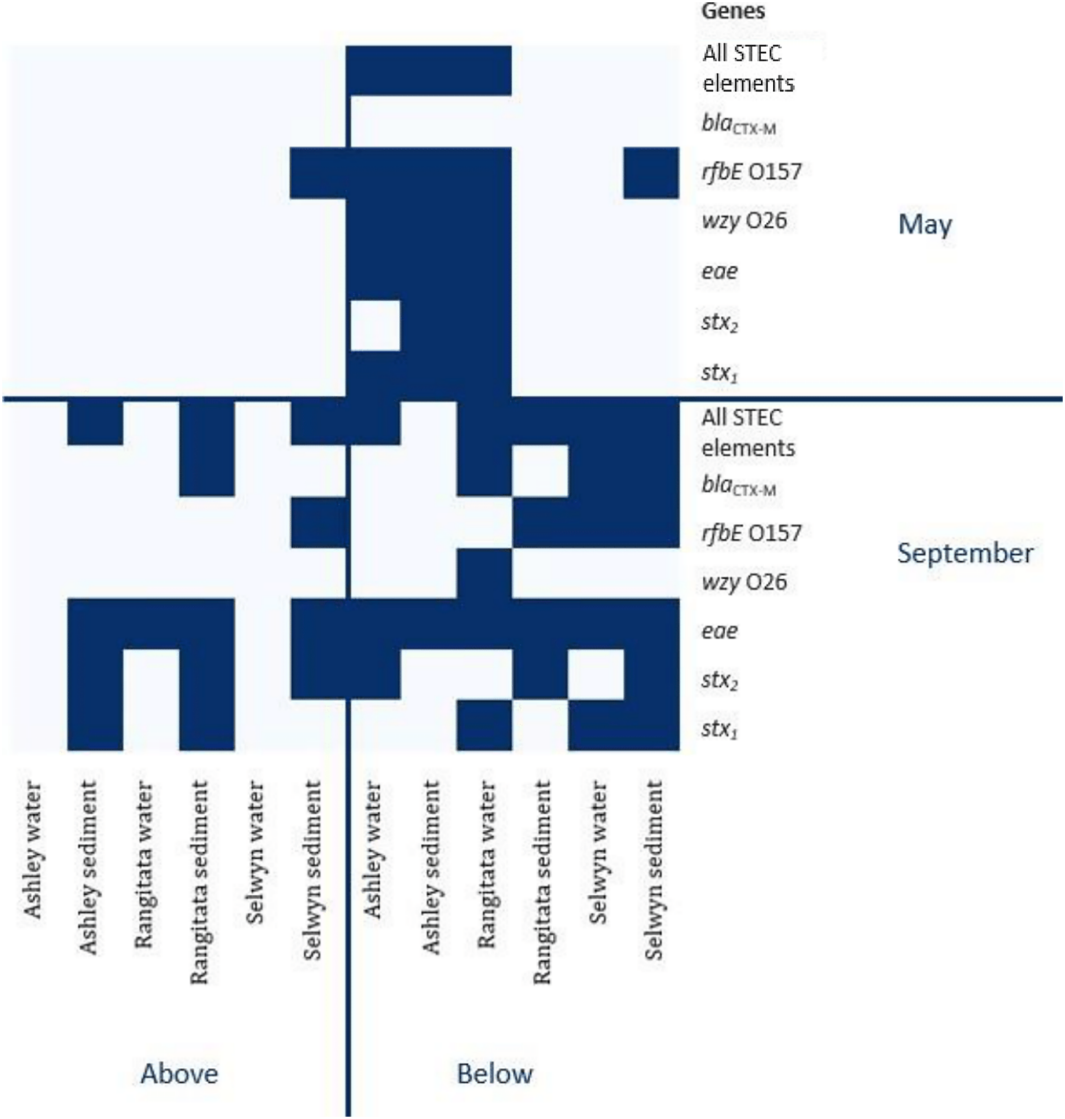
Heatmap of genetic element presence and absence. Heatmap depicting the presence and absence of the six genetic elements associated with human virulence in the Ashley, Rangitata, and Selwyn rivers in May and September of 2018, above and below intensive dairy sites. Locations at which all the elements necessary for human pathogenic STEC are signified by ‘All HP STEC elements’.

In spring the *bla*_CTX-M_ gene was detected in both substrates on the Rangitata and Selwyn rivers and was more frequent ‘below intensive dairy’. However, it was also detected in one sediment sample ‘above intensive dairy’ on the Rangitata. The *stx*_*1*_, *stx*_*2*_ and *eae* STEC virulence genes were detected in all three rivers. The three STEC virulence genes were detected in sediment samples from five of the six sites but only from ‘below intensive dairy’ in the water samples; the *eae* gene alone, was only detected in water ‘above intensive dairy’ from the Rangitata (Fig. 2). In spring samples, the gene associated with the O157 serogroup (*rfbE*) was detected 4 times more often than that associated with the O26 serogroup (*wzy*). In three samples *stx*_*1*_, *stx*_*2*_ and *eae* were all detected (Fig. 2); in one of these the gene associated with the O157 serogroup was present but the genes associated with O157 and O26 were not detected in the remaining two. The *stx*_*1*_ and *eae* genes were detected in two water samples from ‘below intensive dairy’ whereas *stx*_*2*_ and *eae* were detected in three sediment samples, two from ‘below’ and one ‘above intensive dairy’.

## Discussion

Research is limited on the prevalence and distribution of AMR and zoonotic bacteria in New Zealand waterways because growing, isolating, and identifying all potential target organisms from the potential millions contained in a single sample is time consuming and costly. In part, this is because identification through culturing is complicated by diverse metabolic requirements and identifying individual organisms present in a sample would require multiple culture methods (Jaros et al., 2013; Irshad et al., 2016). In studies of ESBLs and STEC strains from ruminant faeces and environmental samples the microbial communities are generally enriched prior to DNA-based testing (Muirhead et al., 2004; Gluckman, 2017). The potential impacts of such enrichment on the microbial communities and the associated detection of zoonotic bacteria are difficult to quantify. In the present study, we examined samples for genes associated with AMR and STEC virulence without an initial enrichment step.

In our analyses the genetic markers indicative of *E. coli* (*uidA*) genes were detected in all three of the rivers sampled. Every sample tested positive for the *uidA* gene, consistent with *E. coli* being isolated at all six sites, from both substrates, and at both sampling times using traditional plating.

Using relatively small sample volumes we were able to detect genes associated with both AMR and human pathogenic STEC. Although all the genes associated with human pathogenic STEC virulence (*stx*_*1*_, *stx*_*2*_ and *eae*) and O serotype (O26 *wzy* and O157 *rfbE*) were present in every river, their presence varied by location, substrate, and season. Most importantly, all the genes necessary for human pathogenic STEC (*e.g*., one toxin gene—*stx*_*1*_ or *stx*_*2*_—and *eae*) were detected within a single sample 11 times and AMR genes were only detected from samples that contained all the genes for human pathogenic STEC. These results suggest that explanations for the distribution of these genes across the landscape are likely to be complex and involve a range of factors.

*Escherichia coli* levels were higher and AMR and STEC associated genes more common at sites below intensive dairy. These results are consistent with previous reports suggesting that agricultural effluent is a major source of faecal contamination in New Zealand waterways (Gluckman, 2017). Faecal bacteria may be transferred from pastures to waterways *via* run-off; carried either directly by the flow or indirectly as a result of adsorption to soil particles (Palmateer et al., 1993; Muirhead et al., 2004; Byappanahalli & Ishii, 2011). In the current study we sampled adjacent to intensive dairy operations but expected microbial communities to vary along each river as the diversity of land use changes downstream. Microbial communities are likely to be most strongly influenced by adjoining land use but will also be influenced by inputs from upstream. For example, the *stx*_*1*_ and *stx*_*2*_ genes were only detected at sites above intensive dairy during spring. One explanation for this may be inputs from smaller farms or non-farm inputs upstream of the reaches sampled. Importantly, the detection limit of the STEC assay at 10^3^ CFU per ml (Anklam et al., 2012) is not so sensitive that a negligible presence of the targeted genetic elements would be over detected. A positive finding suggests that the genetic elements associated with STEC disease in humans were present in the waterways in numbers significant enough to warrant further investigation to ensure public health safety.

The AMR and STEC associated genes occurred at greater frequency in the spring sampling than the autumn sampling. Specifically, the STEC were detected in eight of 12 samples (66%) in the spring and in three of 12 samples (25%) in the autumn whereas the AMR gene was only detected in the spring from two rivers. Detection of the AMR gene during the spring may reflect patterns of antibiotic use on the surrounding farms. Antibiotics are often administered to drying-off cows during winter as well as during spring calving and early milking (Bryan & Hea, 2017), potentially selecting for AMR bacteria in the cow microbiome. Additionally, spring calving likely increases the bacterial load on land bordering these rivers. As calves have poorly developed intestinal biomes, are stressed by weaning, or are removed from their mother prior to receiving colostrum they are prone to colonization by, and heavy shedding of, bacteria, including AMR and STEC strains (Garzio-Hadzick et al., 2010; Gluckman, 2017). In addition to the increased faecal loading on pastures, spring rainfall patterns may lead to higher faecal or other, non-farm related inputs reaching rivers. However, further work including quantification of relevant bacterial genes in water and sediment is required to assess the influence of land-use upon waterways.

In all three of the sampled rivers bacterial colonies indicative of *E*. *coli* were consistently higher in sediments than the water column. This is a common finding frequently attributed to substrate stability; sediments are less mobile than flowing water, therefore a longer-term and more stable habitat for bacteria (Boehm et al., 2009; Fluke, González-Pinzón & Thomson, 2019). The AMR gene was equally likely to be found in sediment or water; but STEC associated genes were more commonly detected in sediment samples. These results are consistent with previous studies that indicate aquatic sediments may act as a store for *E*. *coli* (Anderson, Whitlock & Harwood, 2005; Bryan & Hea, 2017). Given that such stores may persist for months or years and that suspended sediments increase *E*. *coli* levels in the water column, this finding has potentially important implications for monitoring specific pathogenic and CTX-M producing *E*. *coli* strains (Davies-Colley, Valois & Milne, 2018; Weiskerger & Whitman, 2018). Generally, water testing by environment agencies is restricted to the water column. Although recreational use of waterways is discouraged when levels of suspended sediment are high (*e.g*., following precipitation) (Davies-Colley, Valois & Milne, 2018), disturbance of sediments by recreational users is not generally considered. Further work is needed to quantify the pathogens associated with the localized mixing of sediment into the water column by recreational users and to determine whether zoonoses are being underestimated by the sampling of a single microhabitat.

## Conclusions

Monitoring of recreational waterways for human pathogens is a complex but important task. In part this can be attributed to the metabolic diversity of the bacteria themselves and the apparent lack of a relationship between faecal indicator bacteria (*e.g*., counting colonies visually assessed to be *E*. *coli*) and the presence of genes associated with virulence and/or AMR. Moreover, the virulence or AMR status of isolated bacteria are not routinely assessed leading to delays in risk management. Understanding the relationship between pathogenic bacteria and AMR in recreational waterways would require more intensive sampling over a broader geographic range and the inclusion of multiple substrates in sampling protocols. In most cases monitoring is conducted on samples retrieved from the water column. Such samples may not accurately reflect the microbial community that recreational users of the waterway are potentially exposed to. Our results suggest sediments may act as an important reservoir of bacteria and resuspension of these sediments by waterway users could potentially increase exposure to pathogenic strains.

There is a growing appreciation of new technologies that enable the presence and persistence of zoonoses to be monitored without the need for microbial culturing (Palmateer et al., 1993; Pilliod et al., 2019). This represents a fundamental change in our approach to microbial monitoring allowing us to take a holistic view of the riverine environment and improving our ability to protect environmental, animal, and human health. While small, this study is the first step towards understanding zoonoses in waterways at a time when global health is under the microscope.

## Additional Information and Declarations

### Field Study Permissions

The following information was supplied relating to field study approvals (*i.e*., approving body and any reference numbers): Public property, no permit needed.

### Data Availability

The following information was supplied regarding data availability: The data is available in the tables.
